# Mecillinam activity against multidrug-resistant *Shigella sonnei* and *Shigella flexneri*

**DOI:** 10.1128/spectrum.01006-24

**Published:** 2025-02-07

**Authors:** Aleksandra Stefanovic, Leah Gowland, Gordon Ritchie, Colin Lee, Sam Chorlton, Nancy Matic, Victor Leung, Michael Payne, Christopher F. Lowe, Marc G. Romney

**Affiliations:** 1 Division of Medical Microbiology and Virology, St. Paul’s Hospital, Providence Health Care, Vancouver, British Columbia, Canada; 2 Department of Pathology and Laboratory Medicine, University of British Columbia, Vancouver, British Columbia, Canada; 3 Department of Pharmacy, Providence Health Care, Vancouver, British Columbia, Canada; 4 BugSeq Bioinformatics Inc., Vancouver, British Columbia, Canada; Seton Hall University, South Orange, New Jersey, USA

**Keywords:** mecillinam, multidrug resistance, *Shigella*, pivmecillinam, antimicrobial resistance, diarrhea

## Abstract

We performed phenotypic susceptibility testing and whole-genome sequencing to investigate *in vitro* mecillinam activity against multidrug-resistant (MDR) *Shigella*. Ninety-five isolates (53 [56%] *Shigella sonnei* and 42 [44%] *Shigella flexneri*) were included. The MIC_50_ and MIC_90_ were 1 and 2 µg/mL, respectively, by agar dilution, and all zone diameters were ≥15 mm by disc diffusion testing. No known clinically relevant mecillinam resistance mutations (including *cys*B) were detected. The favorable MIC profile and absence of resistance markers make pivmecillinam an agent worth considering for the treatment of infections caused by MDR *Shigella*. Clinically feasible susceptibility testing methods and standardized interpretations for mecillinam and *Shigella* spp. are needed to guide therapy.

**IMPORTANCE:**

Rapidly emerging resistance in *Shigella* species leaves few antibiotic treatment options. The World Health Organization recommends pivmecillinam, a prodrug of mecillinam, for the treatment of *Shigella* infections. However, little is known about the susceptibility of *Shigella* spp. to mecillinam in North America. We performed mecillinam susceptibility testing on our collection of resistant *Shigella* isolates and investigated genetic mechanisms of resistance using whole-genome sequencing. We observed a favorable mecillinam susceptibility profile and a lack of known genetic mechanisms of resistance. However, in the absence of standardized laboratory guidelines for mecillinam susceptibility testing, interpreting susceptibility test results is challenging. We propose that further studies are needed to correlate susceptibility testing data with clinical outcomes, with the aim of establishing standardized clinical breakpoints for *Shigella* spp. and mecillinam.

## OBSERVATION

Multidrug-resistant (MDR) *Shigella* has emerged as a pathogen of global public health concern, leading to increased disease severity among certain populations (1). World Health Organization guidelines recommend ceftriaxone or pivmecillinam for the treatment of MDR *Shigella* infections (2). As ceftriaxone is the only antibiotic widely available for the treatment of MDR shigellosis in North America, challenges arise in parenteral antibiotic administration, including infusion-related complications and additional healthcare costs. Pivmecillinam, an oral prodrug of mecillinam, inactivates penicillin-binding protein 2 (PBP2) of gram-negative bacteria (3). It has been licensed for the treatment of simple urinary tract infections (UTIs) worldwide and salmonellosis in the UK (4). The clinical effectiveness of pivmecillinam has been established for the treatment of shigellosis in children and adults (5–7). In Bangladesh, pivmecillinam has been used to treat drug-resistant shigellosis (8). Little is known regarding mecillinam’s activity against MDR *Shigella* in North America. We performed a phenotypic and genotypic assessment of mecillinam activity against MDR *Shigella* isolates recovered at a tertiary-care hospital laboratory servicing downtown Vancouver, Canada.

Isolates of *Shigella* spp. recovered from stool and blood cultures of adult patients from January 2022 to September 2023 were included in the study. We identified *Shigella* using conventional biochemical methods, Vitek2 ID (bioMérieux,) and Polyvalent Agglutination Sera (Remel). Antimicrobial susceptibility testing to first-line agents (ampicillin, trimethoprim-sulfamethoxazole, and ciprofloxacin), as well as second-line agents (azithromycin and ceftriaxone), was performed as per Clinical and Laboratory Standards Institute (CLSI) M100 (9). Multidrug resistance was defined as resistance to all first-line agents. Mecillinam zone diameters (ZDs) were determined by disc diffusion (DD) testing using 10 µg mecillinam discs (Oxoid). Mecillinam MICs were obtained by agar dilution testing as per CLSI M07 (10). Briefly, previously frozen isolates were inoculated into Mueller-Hinton broth (Oxoid) and adjusted to 0.5 MacFarland turbidity. Muller-Hinton agar dilution plates were prepared in concentrations ranging from 0.03 to 64 µg/mL and used within 24 hours. An inoculum of 10^4^ CFU of organism per spot was deposited with the assistance of an inoculum-replicating device. *Escherichia coli* ATCC25922 and *Enterococcus faecalis* ATCC29212 strains were used as control organisms.

Whole-genome sequencing (WGS) was performed on all isolates for genotyping and identification of genetic determinants of antibiotic resistance (11, 12). DNA was extracted on the MagNA Pure 24 (Roche Diagnostics) and sequenced on the GridION (Oxford Nanopore Technologies) R10.4.1 flowcells with Guppy (version 6.4.6) super accuracy basecalling model. FASTQ files were submitted to BugSeq Bioinformatics (bugseq.com) for assembly and resistance genotyping as previously described (13). Genetic markers conferring ampicillin and ceftriaxone resistance were interrogated. For mecillinam resistance prediction, BLAST searches were performed on the assembled isolate sequences for mutations and deletions in the putative mecillinam resistance genes, including *cysB*, *mrdA, mrdB, mreB, mreC, mreD, rpoB, alaS, gltX, rcsB, rcsC, rcsD, rcsF, yrfF, lon, ppa, thrS, aspS, spoT, argS, ftsZ, cyaA, ubiE, slt,* and *galE,* as described by Thulin et al. (14). The alignments were compared with the reference sequences to detect potential antibiotic resistance mutations.

We constructed a scatterplot and calculated Pearson’s coefficient to measure the relationship between MICs with ZDs (Excel, version 2402). We utilized *t*-test to calculate the two-tailed *P*-value for the ZD difference between *Shigella flexneri* and *Shigella sonnei* isolates, using MedCalc Software Ltd (version 22.018; accessed 18 November 2024).

Ethics approval was waived as isolates were collected and tested as per routine patient care, and mecillinam testing results were not reported for patient care.

**Fig 1:**
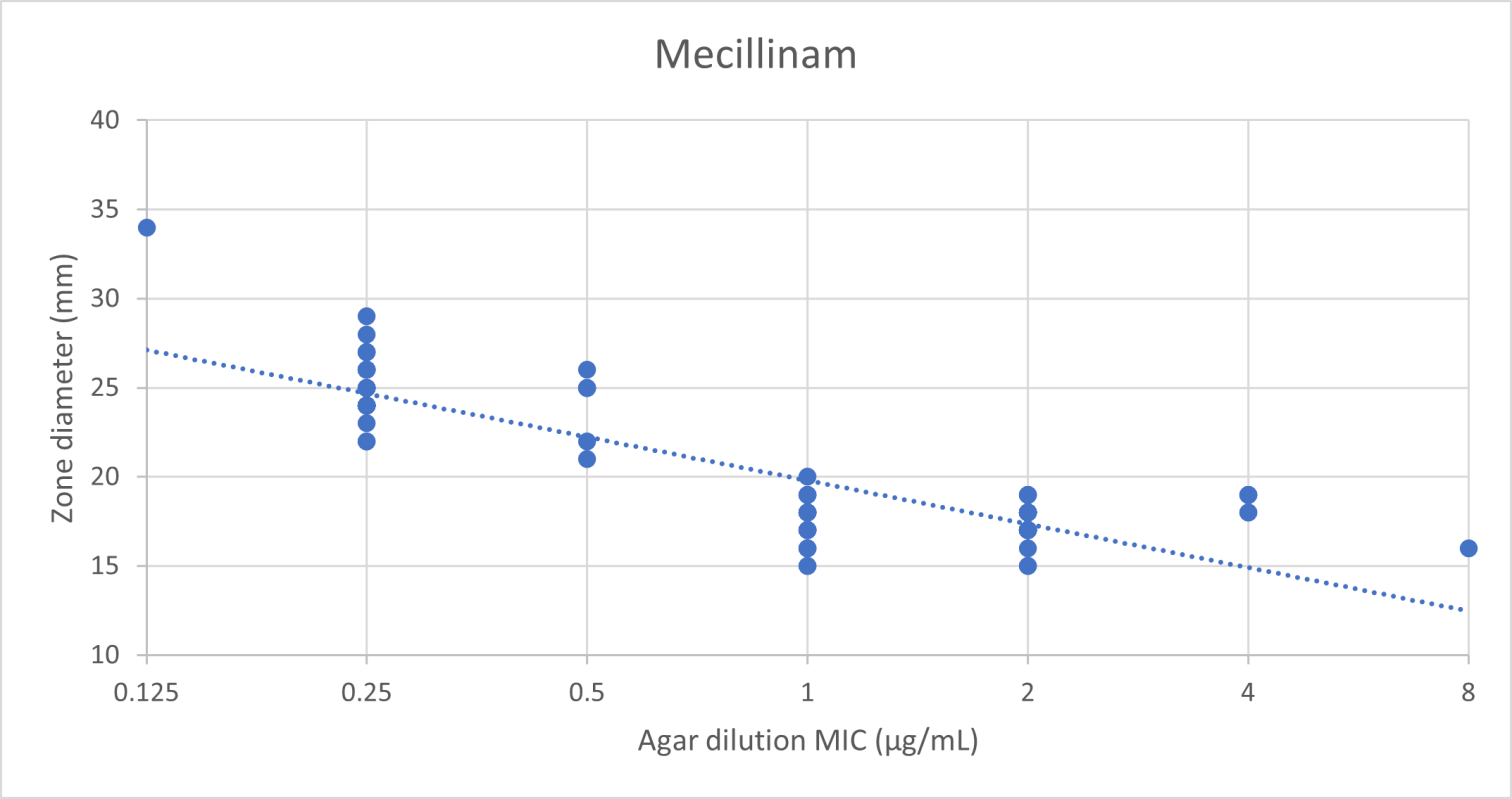
Scatterplot of mecillinam MIC values (agar dilution) versus ZDs (disc diffusion) for all *Shigella* isolates. Pearson’s coefficient *r* = −0.611; *P* < 0.001 and *R*^2^ = 0.731.

Ninety-five *Shigella* isolates (53 *S*. *sonnei* and 42 *S*. *flexneri*) were identified during the study period. Of these, 93 were recovered from stool and 2 from blood cultures. Multidrug resistance was present in 98% and 93% of *S. sonnei* and *S. flexneri* isolates, respectively. Additionally, resistance to azithromycin was detected in 98% of *S. sonnei* and 17% of *S. flexneri* isolates. Both species exhibited near-complete susceptibility to ceftriaxone (98%). Mecillinam MICs and ZDs are shown in Table 1. Mean ZD was higher for the population of *S. flexneri* isolates compared to *S. sonnei* isolates (*t*-test difference 6.2 mm, 95% CI = 5.15–7.33, *P* < 0.0001). Similarly, MIC_50_ was lower for *S. flexneri* than *S. sonnei* isolates at 0.25 µg/mL versus 2 µg/mL, respectively. For control strains, *E. coli* and *E. faecalis* MICs were 0.125 µg/mL (ZD = 29 mm) and ≥64 µg/mL (ZD = 6 mm), respectively, within the expected range. We found a moderate inverse correlation between ZDs and MICs for all included *Shigella* spp. (Pearson’s coefficient *r* = −0.61, *P* < 0.001) (Fig. 1).

**TABLE 1:** Zone diameters and MICs by *Shigella* spp.

Mecillinam	Mean ZD ± SD (mm)	ZD range (mm)	MIC_50_ (µg/mL)	MIC_90_ (µg/mL)	MIC range (µg/mL)
*Shigella sonnei (n =* 53)	17.8 ± 2.21	15–28	2	4	0.25–4
*Shigella flexneri (n =* 42)	24.0 ± 3.12	16–34	0.25	1	0.125–8
All *Shigella* spp. (*n =* 95)	20.6 ± 4.09	15–34	1	2	0.125–8

Of the 53 *S*. *sonnei* isolates, 98% belonged to the 3.6.1.1.2 (CipR.MSM5) genotype, while a single isolate was assigned to genotype 3.7.16 (Global III). Of the 42 *S*. *flexneri* isolates, serotype 2a accounted for 88% of isolates, 1c (7a) for 7% of isolates, while serotypes 1b and X accounted for 2% each. β-lactamase genes identified by WGS in *S. sonnei* isolates included *bla_TEM-1B_* and *bla_C_*_TX-M-15_ in 52 (98%) and 1 (2%) of isolates, respectively. Among *S. flexneri* isolates, *bla*_OXA-1_, *bla_TEM-1_*, *bla_TEM-40_*, *bla_DHA-1_*, and *bla_CTX-M-15_* were found in 40 (95%), 5 (12%), 3 (7%), 1 (2%), and 1 (2%) of isolates, respectively. On interrogation for genes known to confer mecillinam resistance, no resistance mutations were detected, including the absence of *cysB* mutations.

Although mecillinam MICs (≤8 µg/mL) and ZDs (≥15 mm) for our collection of MDR *Shigella* isolates fell within the susceptible range for *Enterobacterales* (urine) by EUCAST, we were unable to interpret these results, as no clinical breakpoints (CBs) for *Shigella* species and mecillinam in non-urinary sites have been established (15). For the majority of our isolates, MICs were below the achievable serum concentration of 2.5 and 6.25 µg/mL after doses of 400 and 800 mg of pivmecillinam, respectively (16). However, human pharmacokinetic studies of mecillinam distribution and concentration in the gastrointestinal tract are sparse (17). Pivmecillinam administration in healthy volunteers has been associated with a decrease in intestinal numbers of *E. coli,* but data on its effect on *Shigella* are lacking (18). Furthermore, pivmecillinam has been licensed in the UK for the treatment of acute typhoid fever and *Salmonella* carriers (19). As pivmecillinam is recommended for the treatment of MDR and extensively drug-resistant (XDR) *Shigella*, the establishment of CBs is much needed to guide therapy (2, 20).

Agar dilution is a reference method according to CLSI and EUCAST; however, it is labor-intensive, subject to inoculum-dependent variability, mecillinam degradation, and not available in most clinical laboratories (21). In contrast, DD testing is simple to perform and could serve as a feasible alternative method. We found a moderate correlation between ZDs and MICs. Others have shown an excellent correlation of ZDs with MICs for *Enterobacterales* (21, 22). However, Barry (21) cautioned when interpreting “borderline” zone sizes (14–16 mm) as these can be discordant. Additional *Shigella* strains, including mecillinam-resistant ones, should be included in the comparison of ZDs with MICs.

As mecillinam remains stable in the presence of narrow-spectrum β-lactamases, the presence of predominantly TEM-1 and OXA-1 β-lactamases with resistance to ampicillin did not lead to elevated MICs to mecillinam (3). There were only two isolates harboring CTX-M-15 in our collection, with low mecillinam MICs of 0.25 µg/mL. Mecillinam also retains activity against many ESBL- and AmpC-harboring bacteria, while resistance has only been described with certain CTX-M-type enzymes (i.e., CTX-M-127 and CTX-M-215) (23–25). Since most β-lactamases do not affect mecillinam activity, other putative genotypic mechanisms of resistance have been explored in *E. coli* (14, 25). A number of genes can confer mecillinam resistance when mutated in a laboratory; however, only a few, such as *cysB* mutations, have resulted in resistance in clinical isolates from patients with *E. coli* UTIs (14, 25). Cysteine biosynthesis is inactivated in c*ysB* mutants, resulting in an oxidative stress response that upregulates PBP1B and LpoB proteins and bypasses the need for functional PBP2 (26). Mutations in *cysB* conferring mecillinam resistance have also been described in *Salmonella enterica* serovar Typhimurium (27). We found no evidence for the presence of any of the 26 putative resistance mutations interrogated, including none of the more common *cysB* mutations (14). Interestingly, among our isolates, there was a species-specific difference in susceptibility, with *S. flexneri* exhibiting lower MICs and larger ZDs compared to *S. sonnei*. This phenomenon has not been previously reported and requires further study.

Pivmecillinam is not currently in use clinically in our patient population, which may impact the prevalence of phenotypic resistance and associated mutations. Although directed use of pivmecillinam for *E. coli* UTIs has not resulted in increased resistance in Europe, the indiscriminate use of pivmecillinam could lead to the development of resistance, as reported in Bangladesh where it is used for empirical treatment of shigellosis (8, 28).

Limitations of our study include the predominance of a single serotype among *S. sonnei* and *S. flexneri* isolates leading to lower strain diversity. Additional strains (including XDR *Shigella*) should be tested for mecillinam susceptibility. We did not have access to mecillinam-resistant strains of *Shigella*. Instead, we used an *E. faecalis* control strain with intrinsic mecillinam resistance, which exhibited MIC ≥ 64 µg/mL and no zone of inhibition around the disc. Although genotypic mechanisms of mecillinam resistance specific to *Shigella* species have not yet been elucidated, no currently known clinically relevant mutations conferring resistance in *E. coli* have been detected. Further work is needed to explore other potential mechanisms of mecillinam resistance in *Shigella* spp.

We demonstrated relatively low mecillinam MICs among MDR *Shigella* isolates and the absence of known genetic resistance markers, suggesting that pivmecillinam is worth considering for the treatment of infections due to MDR *Shigella*. The moderate correlation observed between ZDs and MICs warrants further evaluation, including testing a wider range of isolates. Further research is needed to establish CBs for *Shigella* spp., incorporating *in vitro* testing, pharmacokinetic/pharmacodynamic parameters, and clinical outcomes.

## Data Availability

Sequencing data have been submitted to GenBank and is available through Bioproject links at NCBI (ncbi.nlm.nih.gov). For *Shigella flexneri* isolates, the Bioproject is PRJNA1192303. For *S*. *sonnei*, the Bioprojects are PRJNA1139194 and PRJNA1019784.
